# A Possible Cause for the Differential Expression of a Subset of miRNAs in Mesenchymal Stem Cells Derived from Myometrium and Leiomyoma

**DOI:** 10.3390/genes13071106

**Published:** 2022-06-21

**Authors:** Mariangela Di Vincenzo, Concetta De Quattro, Marzia Rossato, Raffaella Lazzarini, Giovanni Delli Carpini, Andrea Ciavattini, Monia Orciani

**Affiliations:** 1 Department of Clinical and Molecular Sciences-Histology, Università Politecnica delle Marche, 60126 Ancona, Italy; m.divincenzo@pm.univpm.it (M.D.V.); r.lazzarini@univpm.it (R.L.); m.orciani@univpm.it (M.O.); 2 Department of Biotechnology, University of Verona, 37134 Verona, Italy; concetta.dequattro@univr.it (C.D.Q.); marzia.rossato@univr.it (M.R.); 3 Clinic of Obstetrics and Gynecology, Department of Clinical Sciences, Università Politecnica delle Marche, 60126 Ancona, Italy; g.dellicarpini@univpm.it

**Keywords:** miRNAs, progenitor cells, amniotic fluid, leiomyoma, myometrium, divergent cell commitment, linear dysregulation

## Abstract

The aetiology of leiomyoma is debated; however, dysregulated progenitor cells or miRNAs appear to be involved. Previous profiling analysis of miRNA in healthy myometrium- (M-MSCs) and leiomyoma- (L-MSCs) derived mesenchymal stem cells (MSCs) identified 15 miRNAs differentially expressed between M-MSCs and L-MSCs. Here, we try to elucidate whether these differentially regulated 15 miRNAs arise as a conversion of M-MSCs along the differentiation process or whether they may originate from divergent cell commitment. To trace the origin of the dysregulation, a comparison was made of the expression of miRNAs previously identified as differentially regulated in M-MSCs and L-MSCs with that detected in MSCs from amniotic fluid (considered as a substitute for embryonic cells). The results do not allow for a foregone conclusion: the miRNAs converging to the adherens junction pathway showed a gradual change along the differentiation process, and the miRNAs which coincided with the other three pathways (ECM-receptor interaction, TGFβ and cell cycle) showed a complex, not linear, regulation and, therefore, a trend along the hypothetical differentiation process was not deduced. However, the role of miRNAs appears to be predominant in the onset of leiomyoma and may follow two different mechanisms (early commitment; exacerbation); furthermore, miRNAs can support the observed (epigenetic) predisposition.

## 1. Introduction

Uterine fibroids, also called leiomyomas, are the most common benign gynecologic tumors affecting women during their reproductive age, with an incidence directly related to age [1]. Epigenetic mechanisms, gene mutations, chronic inflammation, disrupted controls in progenitor cells, and dysregulation of miRNAs have been all evaluated as potential causes; however, their aetiology has not been fully clarified, [2,3].

Previously [4,5], we demonstrated that mesenchymal stem cells (MSCs) isolated from leiomyomas (L-MSCs) and normal myometrium (M-MSCs) can diversely sustain acute and chronic inflammation promoting a microenvironment suitable for leiomyoma onset; additionally, out of 2646 miRNAs, only 15 miRNAs displayed significantly altered expression between leiomyoma and normal myometrium, supporting the hypothesis that leiomyoma derives from the disruption of specific cellular mechanisms in progenitor cells.

A population of MSCs occurs in almost all human tissues [6]. According to the criteria outlined by Dominici [7], MSCs must be plastic adherent, positive for CD73, CD90, and CD105 and negative for HLA-DR, CD14, CD19, CD34, and CD45 and be able to differentiate towards oste-, chondro- and adipogenic lineages. By fulfilling these criteria, mesenchymal cells derived from adult tissues exhibit tissue-specific features that become even more characteristic during differentiation [8]. Among mesenchymal stem cells, amniotic fluid MSCs (AF-MSCs) are of particular interest, as they express both adult and embryonic cell markers and are therefore considered an intermediate stage between embryonic and adult cells [9]. In a theoretical line of increasing differentiation, the first should be the embryonic cells, followed by AF-MSCs and, finally, by MSCs derived from adult tissues, such as M- and L-MSCs.

**Figure 1:**
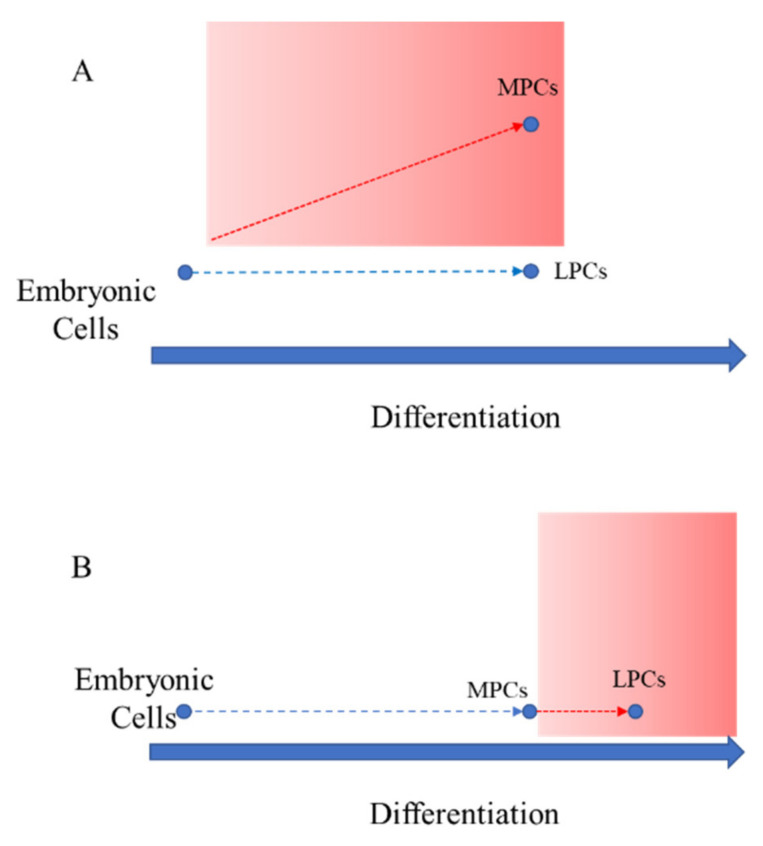
Two different hypotheses for the origin of miRNAs differentially regulated between M-MSCs and L-MSCs. (**A**) M-MSCs and L-MSCs are the results of divergent differentiation from embryonic stem cells; (**B**) embryonic stem cells physiologically differentiate in M-MSCs, and further pathological differentiation produces L-MSCs. The red areas indicate the spectrum of the disease. The red arrows show the pathological differentiation.

miRNAs are switchers able to modulate cell fate by turning on/off specific gene targets, and their aberrant expression can proportionally influence these critical processes leading to various pathological outcomes [10,11]. We recently demonstrated that 15 miRNAs are differentially expressed between MSCs from leiomyoma and healthy myometrium, but we do not know the mechanisms underlying these differences nor their origin. In particular, when do these alterations occur? Do they arise directly from embryonic cells, i.e., differential miRNA expression leads early to different MSCs in normal myometrium and fibroids (as in hypothesis A, Figure 1A)? Alternatively, do they arise later during the last steps of the differentiation process, i.e., embryonic cells produce normal M-MSCs and these, under the control of dysregulated miRNAs, acquire pathological features which give rise to L-MSC (as in hypothesis B, Figure 1B)? Since the use of embryonic cells to answer these research questions is forbidden, AF-MSCs were alternatively used in this study as a substitute for embryonic cells, as it maintains a lower degree of differentiation than MSCs derived from adult tissues and therefore can be used to follow the onset of these alterations during the differentiation process. AF-MSCs are considered as the starting point in the differentiation process and the expression of the 15 dysregulated miRNAs will be compared to those observed in M- and L-MSCs.

## 2. Materials and Methods

### 2.1. Tissue Collection

Amniotic fluid (AF) samples (*n* = 9), 3 mL, were obtained by amniocentesis after the 16th week of pregnancy for routine prenatal diagnosis. Gestational age (GA) was determined by ultrasonic measurements of the biparietal diameter and length of the fetus’s femur. AF was collected by ultrasound-guided transabdominal puncture. The indications were advanced maternal age (34–36) and the cytogenetic analyses revealed normal karyotypes.

Healthy and fibrotic myometrium samples (*n* = 12), 3 mm^2^ in size, were obtained from women of childbearing age (range, 30–35 years) undergoing myomectomy for symptomatic leiomyomas.

All patients provided their written informed consent to participate in the study, which was approved by the institutional ethics committees (2016-0360OR) and conducted in accordance with the Declaration of Helsinki.

Cells were isolated, cultured, and characterized as previously described [4,5,12,13].

Briefly, the solid samples were first mechanically minced into small pieces whereas AF samples were directly centrifuged. The pellets were resuspended and transferred into 6-well plates with MSC growth medium (MSCGM; Lonza-Bioscence, Basel, Switzerland) suitable for the growth of undifferentiated stem cells. The samples were incubated at 37 °C and 5% carbon dioxide. Morphology was assessed by phase-contrast microscopy (Leica DM IL; Leica Microsystems GmbH, Wetzlar, Germany). Vitality and proliferation rate were examined using an automated cell counter (Countess; Invitrogen, Milan, Italy). After sample collection, the cells were characterized by testing the minimal criteria identified by Dominici [7] for mesenchymal definition as previously described [14,15,16].

For immunophenotyping, 2.5 × 10^5^ cells at the 3rd passage were stained for 45 min with fluorescein isothiocyanate (FITC)-conjugated antibodies (Becton-Dickinson, NJ, USA) against HLA-DR, CD14, CD19, CD34, CD45, CD73, CD90, and CD105. Furthermore, CD9 expression was evaluated in MSCs and fibroblasts (FBs) obtained from the same tissues since it is considered a distinctive marker between MSCs and FBs [17]. Differentiation into osteocytes, chondrocytes and adipocytes was assessed using STEMPRO^®^ Osteogenesis, Chondrogenesis, and Adipogenesis Kits (GIBCO, Invitrogen), respectively. Cells cultured in DMEM/F-12 with 10%FBS were used as negative controls.

Osteogenic differentiation was assessed by Alizarin Red and Alkaline phosphatase (ALP) stainings; adipogenic differentiation was tested by Oil Red staining. For chondrogenesis, cells were grown in a pellet culture system, and the sections were exposed to a Safranin-O solution. Fibroblasts obtained from the same tissues were used to compare the differentiation efficiency.

### 2.2. miRNA Profiling

Total RNA was extracted in triplicate using Norgen Total RNA Kit (Norgen, Biotek Corporation, Thorold, ON, Canada) from a pool of mixed cells obtained from the twelve cultures of M-MSCs or of L-MSCs and from the nine cultures of AF-MSCs in triplicate. RNA purity and amounts were measured using a NanoDrop Spectrophotometer (NanoDrop Technologies, INK, Wilmington, DE, USA), whereas RNA integrity (RINA ≥ 8.0) was assessed using an RNA 6000 Nano Kit (Agilent Technologies, CA, USA). miRNA-sequencing libraries were generated with the QiAseq miRNA kit (QIAGEN, Hilden, Germania), assessed by capillary electrophoretic analysis with the Agilent 4200 Tape station and sequenced using 1 × 75 bp-reads on an Illumina NextSeq500 generating about 8 million fragments per sample.

### 2.3. Bioinformatics Analysis

Starting from raw FASTQ files, the quality of reads obtained from each sample was assessed using FastQC software (v.0.10.1) [18] , adapters were trimmed and reads with length < 18 bp or >27 bp were filtered out with cutadapt (1.16). Filtered reads were aligned to human hairpin microRNAs available in the miRBase [19] using SHRiMP2 (v2.2.3. http://compbio.cs.toronto.edu/shrimp/, accessed on 27 April 2020) with the “mirna” mode activated. The number of Unique Molecular Identifiers (UMI) mapped reads were counted for each mature miRNA. Differential miRNA expression analysis between L-MSCs, M-MSCs, and AF-MSCs was performed with DESeq2 (v 1.16.1, https://support.bioconductor.org/, accessed on 27 April 2020). The expression of microRNAs was normalized as CPM (counts per million reads mapped) and subsequently the 15 miRNAs previously identified as differentially expressed between M-MSCs and L-MSCs were clustered by an unsupervised hierarchical clustering using Spearman rank correlation and the average linkage method.

### 2.4. miRNAs Targets Analysis

To identify molecular pathways potentially altered by the expression of single or multiple miRNA, Diana mir-PathSoftware was used [20]. This web-based application performs enrichment analysis of numerous miRNA target genes comparing each set of miRNA targets to all known KEGG (Kyoto Encyclopedia of Genes and Genomes, Kyoto, Japan) pathways.

The input dataset enrichment in each KEGG pathway is represented by the negative natural logarithm of the P value (−lnP).

In addition, to partially compensate for the lack of functional analysis, the mirTarBase [21] software was used to find validated targets of selected miRNAs and the STRING (Search Tool for the Retrieval of Interacting Genes/Proteins) database [22] to construct a protein−protein interaction network; proteins have been included in the analysis only if directly targeted by at least one miRNA in each KEGG pathways.

## 3. Results

**Figure 2:**
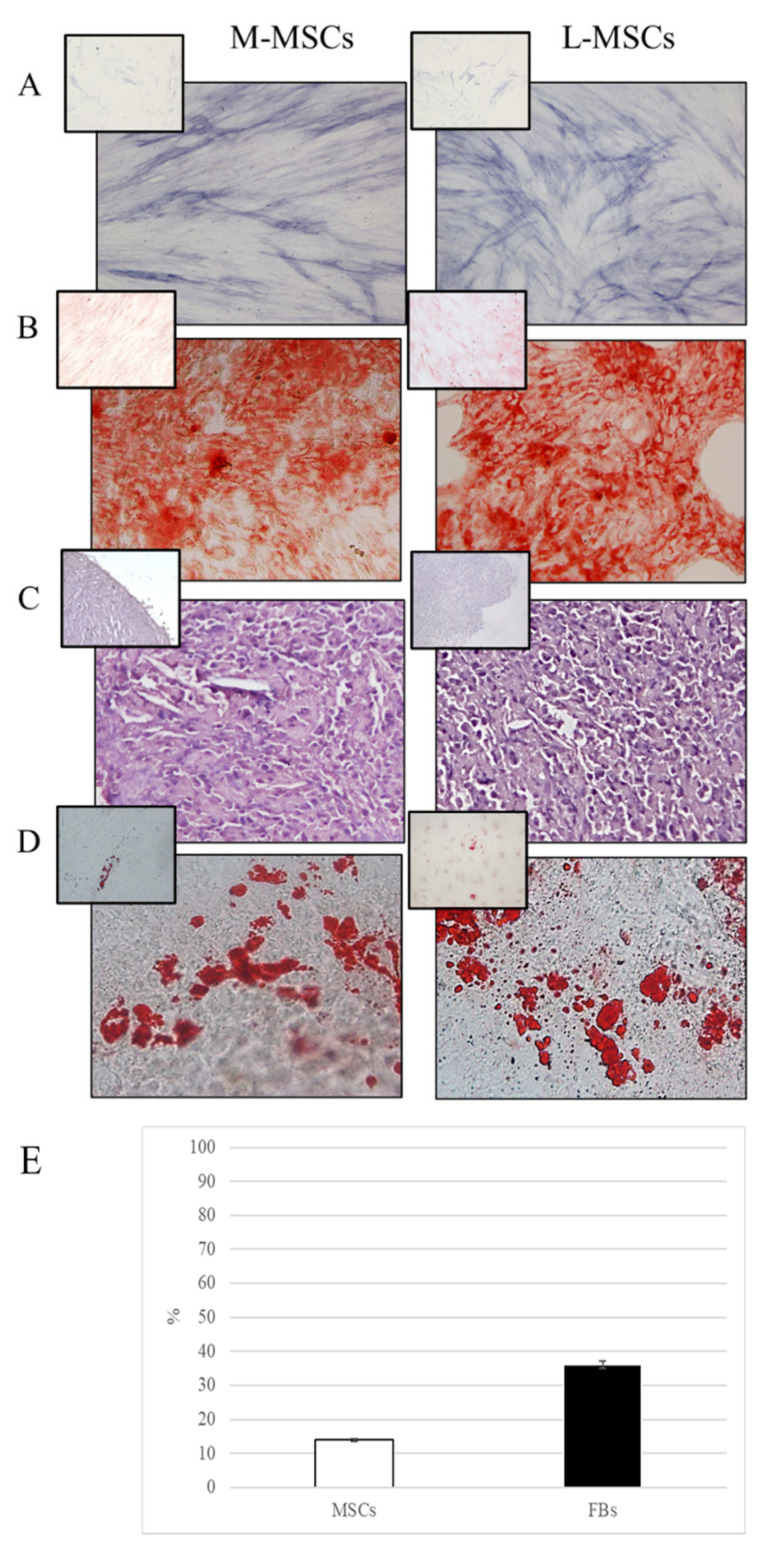
Multilineage differentiation of MSCs from myometrium (M-MSCs) and leiomyoma (L-MSCs) and relative fibroblasts (in the inserts). Representative images of osteogenic differentiation assessment by ALP reaction (**A**) and Alizarin red staining (**B**); chondrogenic differentiation by Safranin-O staining (**C**); adipocyte differentiation by Oil red staining (**D**). Percentage of CD9 positive cells after flow cytometric analysis (E). Data are presented as mean ±SD of the 12 samples of L- and M-MSCs and related FBs.

M-MSCs, L-MSCs, and AF-MSCs, respectively isolated from healthy myometrial, fibroid tissue, and amniotic fluids, met the three criteria established by the International Society for Cellular Therapy (ISCT) for the identification of human mesenchymal stem cells [7]. Cells were adherent to plastic under standard culture conditions, strongly positive for CD105, CD73, and CD90 and negative for CD45, CD34, CD14, CD19, and HLA-DR and were able to differentiate towards mesenchymal lineages in vitro (Figure 2A–D). FBs isolated from the same tissues were induced to differentiate but their ability, as shown in the inserts, was notably inferior to MSCs. Finally, CD9 expression was evaluated by flow cytometry and the percentage of positive cells was 14% and 36% for MSCs and FBs, respectively, confirming the undifferentiated nature of isolated cells (Figure 2E).

**Figure 3:**
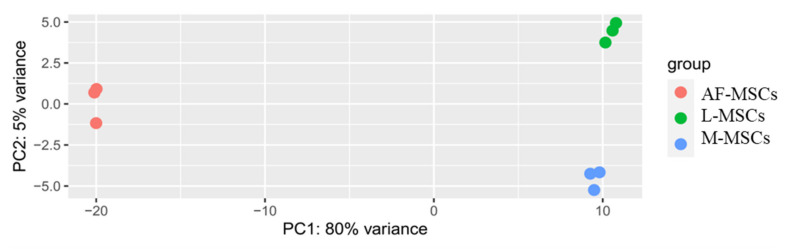
Principal Component Analysis (PCA) shows the variance of samples analysed in the study, based on the full miRNA profile (2646 expressed miRNAs). The analysis was conducted using the DESeq2 package and the values on each axis represent the percentages of variation explained by the principal components. PC1, principal component 1; PC2, principal component 2.

RNA obtained from the three cellular pools was used for miRNA profiling by RNA-sequencing [5]. Principal Component Analysis shown in Figure 3 is based on more than 2600 miRNAs expressed in samples and reveals that M-MSCs and L-MSCs exhibit a more similar miRNA expression profile than AF-MSCs, highlighting that MSCs from amniotic fluid are more distant, and so are dissimilar to those of adult tissues.

**Figure 4:**
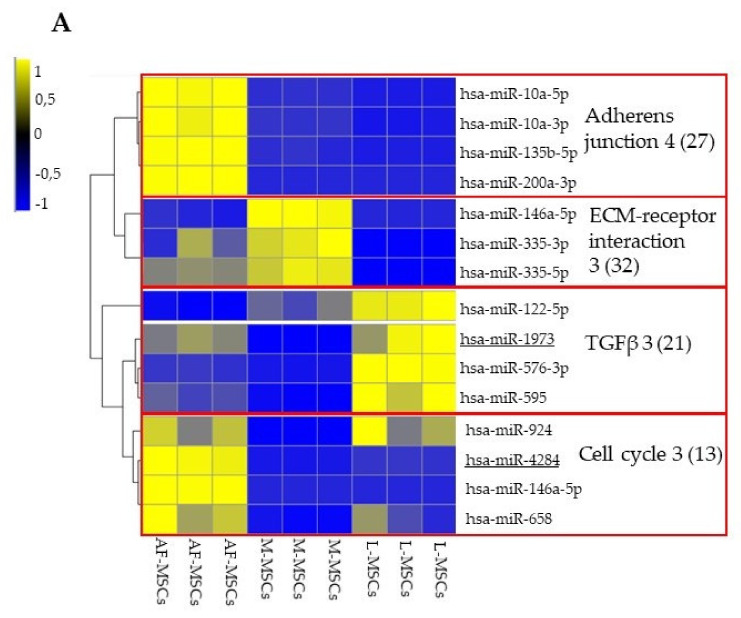

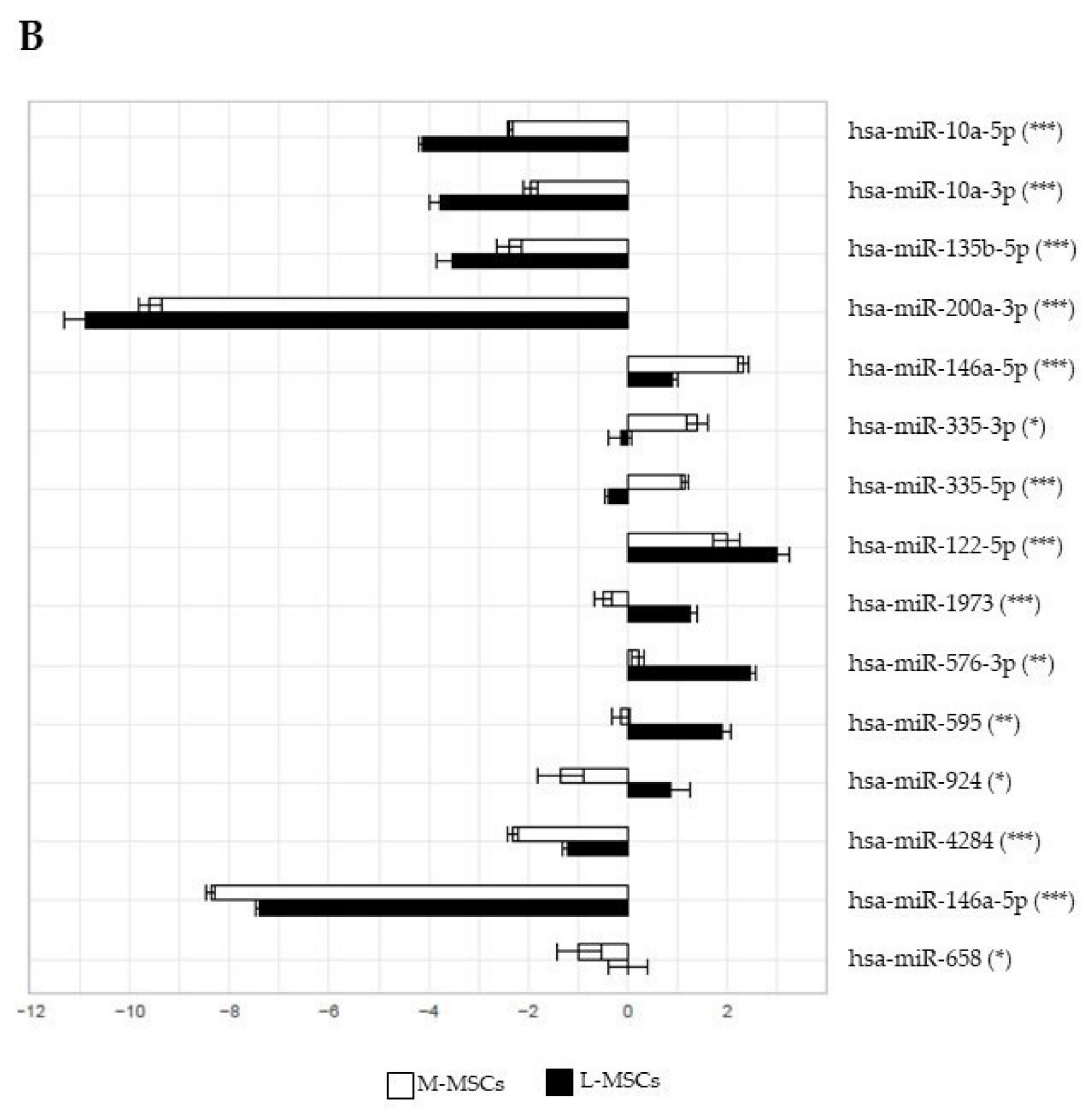
Heat map and expression fold change of 15 miRNAs previously identified as differentially expressed between M-MSCs and L-MSCs based on next generation sequencing analysis. (**A**) Each row represents a different miRNA, and each column represents one sample from the AF-MSCs (*n* = 9), M-MSCs (*n* = 12) or the L-MSCs (*n* = 12) RNA pools. The clusters were obtained by unsupervised hierarchical clustering using Spearman rank correlation and the average linkage method. The key colour illustrates the normalized expression levels (z-scores of normalized counts-cpm) of miRNAs in all samples. The yellow to blue gradient indicates a higher to lower expression. In the right column, the KEGG pathways identified by DIANA-miRPath software as targets of the analysed miRNAs. In brackets, the number of targeted genes per pathways. Underlined miRNAs are not convergent in the indicated KEGG pathway. (**B**) Expression fold changes (log2-transformed) of the 13 selected miRNAs in M-MSCs and L-MSCs as compared to AF-MSCs. Adjusted *p* value < 0.05: *** both comparisons, ** only L-MSCs, * only MSCs.

Subsequently, we recovered the expression of the 15 miRNAs previously found dysregulated in M-MSCs with respect to L-MSCs [5], hsa-miR-10a-3p; hsa-miR-10a-5p; hsa-miR-122-5p; hsa-miR-135b-5p; hsa-miR-146a-5p; hsa-miR-146b-5p; hsa-miR-200a-3p; hsa-miR-335-3p; hsa-miR-335-5p; hsa-miR-576-3p; hsa-miR-595; hsa-miR-658; hsa-miR-924; hsa-miR-1973; and hsa-miR-4284. Clustering analysis sub-grouped the 15 miRNAs into four main clusters based on their level of expression in the cell type analyzed (Figure 4A).

The clustered miRNAs were analyzed by Diana mir- Path Software to identify the related KEGG pathways.

All miRNAs belonging to the first cluster converged to the adherens junction pathway (4 converging miRNAs, hsa-miR-10a-5p, hsa-miR-10a-3p, hsa-miR-135b-5p, and hsa-miR-200a-3p, for a total of 27 target genes); all three miRNAs of the second cluster focused to ECM-receptor interaction pathway (3 converging miRNAs, hsa-miR-146b-5p, hsa-miR-335-3p, and hsa-miR-335-5p, for a total of 32 target genes); for the third cluster, the analysis identified the TGFβ signaling pathway (3 converging miRNAs, hsa-miR-122-5p, hsa-miR-576-3p, and hsa-miR-595, for a total of 21 target genes) with the exclusion of hsa-miR-1973, for which no correlated pathways have been found; finally, three of the four miRNAs grouped in the last cluster merged into the cell cycle pathway (3 converging miRNAs, hsa-miR-924, hsa-miR-146a-5p, and hsa-miR-658, for a total of 13 target genes).

The miRNAs converging to the four pathways (hsa-miR-1973 and hsa-miR-4284 were excluded from the analysis as they did not converge in the pathways identified for their cluster) were differentially expressed in M-MSCs and L-MSCs compared to AF-MSCs and 8/13 of them (hsa-miR-10a-5p; hsa-miR-10a-3p; hsa-miR-135b-5p; hsa-miR-200a-3p; hsa-miR-146b-5p; hsa-miR-335-5p; hsa-miR-122-5p; and hsa-miR-146a-5p) showed a significant difference in both cell types compared to amniotic progenitors (Figure 4B, miRNAs marked with ***). In detail, the four miRNAs (hsa-miR-10a-3p; hsa-miR-10a-5p; hsa-miR-135b-5p; and hsa-miR-200a-3p) that converge at the KEGG pathway “adherens junction” were significantly downregulated in M-MSCs and mainly in L-MSCs compared to AF-MSCs; the three miRNAs (hsa-miR-146b-5p; hsa-miR-335-3p; and hsa-miR-335-5p) targeting genes related to ECM-receptor interaction pathway showed a significant increase in M-MSCs compared to AF-MSCs, whereas their expression was variable in L-MSCs versus AF-MSCs. The expression of the three miRNAs (hsa-miR-122-5p; hsa-miR-576-3p; hsa-miR-595) involved in the TGFβ signaling pathway was significantly higher in L-MSCs than in AF-MSCs, whereas it did not change in 2 of 3 miRNAs (hsa-miR-595; hsa-miR-576-3p) in M-MSCs compared to AF-MSCs; finally, all three miRNAs (hsa-miR-924; hsa-miR-146a-5p; hsa-miR-658) related to cell cycle were significantly downregulated in M-MSCs and only one (hsa-miR-146a-5p) in L-MSCs compared to AF-MSCs.

In the absence of direct functional analysis and in the attempt to strengthen the putative link between differentially expressed miRNAs and altered KEGG pathways, identification of validated protein targets and the evaluation of the protein–protein interaction (PPI) networks were performed using mirTarBase and STRING, respectively.

**Figure 5:**
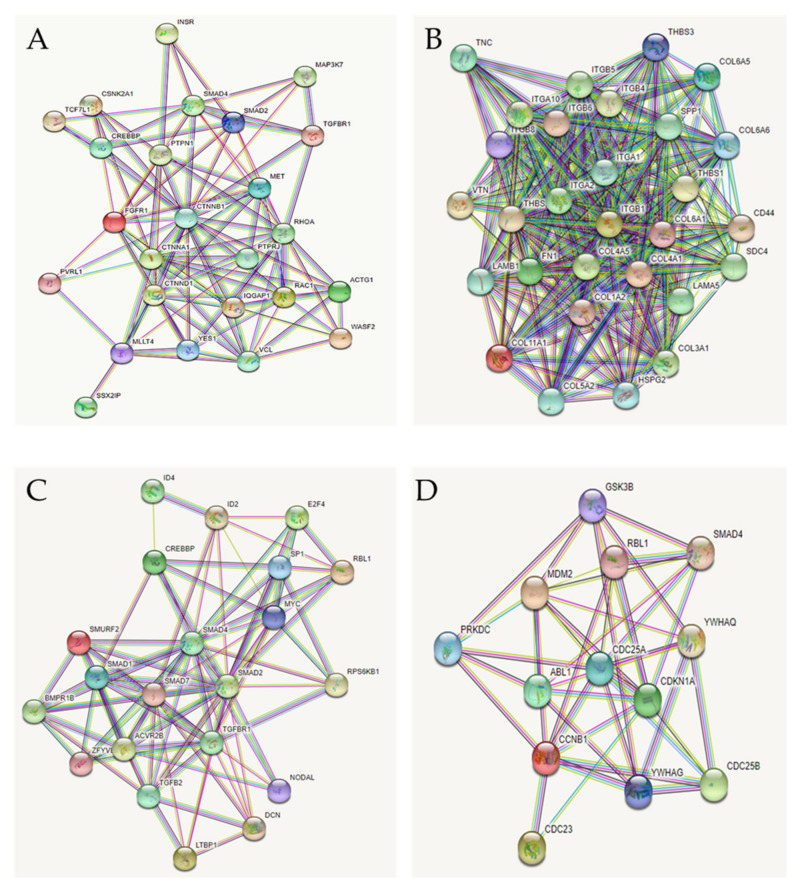
Protein−protein interaction (PPI) network. PPI network has been generated by STRING (Search Tool for the Retrieval of Interacting Genes/Proteins) based on the targeted proteins involved in (**A**) the adherens junction; (**B**) the ECM-receptor interaction pathway; (**C**) the TGFβ; and (**D**) the cell cycle pathway. Nodes are proteins. The thickness of the line is proportional to the strength of the interaction between 2 proteins.

The validated protein targets are listed in Table 1. All proteins are effectively related to the classified KEGG pathways; the PPI networks clearly show the close connection among the proteins enforcing that, even if mostly putative, these targets are specific and highly interconnected (Figure 5).

## 4. Discussion

The expression of 15 miRNAs previously identified as differentially regulated between M-MSCs and L-MSCs [5] was compared with that observed in AF-MSCs in the attempt to elucidate whether dysregulation of the 15 miRNAs occurs: (i) as a result of an early commitment of undifferentiated cells (Figure 1A) or (ii) as an exacerbation of the physiological process of differentiation from M-MSCs to L-MSCs (Figure 1B). AF-MSCs, as a result of their intermediate status between embryonic and adult MSCs, have been considered in this study as a substitute for embryonic cells. However, the regulation of the expression level of the miRNAs across the cell types analysed did not provide conclusive evidence.

The cells isolated from healthy and fibrotic uterine tissues as well as from amniotic fluids were firstly characterized according to the minimal criteria established by the International Society for Cellular Therapy (ISCT) for the identification of human mesenchymal stem cells. In each case, the criteria was met.

After characterization, the choice of AF-MSCs as the most undifferentiated cells, which were used in this study as a substitute for embryonic ones, was validated [9]. Consistent with our hypothesis, AF-MSCs grouped separately from M-MSCs and L-MSCs, whereas the M-MSCs and L-MSCs grouped closer to each other. The expression of the 15 miRNAs in AF-MSCs represents the starting point and will be compared with that detected in M- and L-MSCs.

The heat map and the histogram showed that the expression of the majority of the 15 miRNAs was notably different between AF-MSCs and L-MSCs/M-MSCs (mean of absolute FC 2.6 ± 2.8). The clustering analysis followed by DIANA mir-Path investigation allowed for the identification of four KEGG pathways related to miRNA-clusters defined based on expression: adherens junction, ECM-receptor interaction, TGFβ signaling, and cell cycle. Although the miRNAs targets were not functionally validated, their role was confirmed by mirTarBase and STRING. mirTarBase reported previously validated targets of the selected miRNAs which belong to the identified KEGG pathways. At the same time, STRING showed strong connections among identified putative proteins.

The trend of expression across the cell types of each identified pathway was evaluated in order to determine which hypothesis was more consistent (A, early commitment or B, exacerbation). All four miRNAs (hsa-miR-10a-3p; hsa-miR-10a-5p; hsa-miR-135b-5p; and hsa-miR-200a-3p) converging to the adherens junction pathway showed a stepwise manner along the differentiation process. Within the other three pathways (ECM-receptor interaction, TGFβ, and cell cycle), the miRNAs showed different trends and, therefore, a progressive increase or decrease in expression from AF-MSCs to L-MSCs, i.e., along the hypothetical differentiation process, could not be deduced.

Further, L-MSCs expressed the lowest miRNAs values related to the adherens junction pathway and, most likely as a consequence, the highest level of targeted proteins. As expected, the expression of adhesion molecules is higher in cells residing in connective tissues (where fibroblasts/myofibroblasts develop strong bonds to ECM) than in AF-MSCs [23]. Although the increased expression of proteins related to adherens junction from AF-MSCs to M-MSCs must be considered physiological, its additional shift towards L-MSCs may be connected to the onset of leiomyoma [24,25].

Regarding the ECM-receptor interaction pathway, miRNAs (hsa-miR-146b-5p; hsa-miR-335-3p; and hsa-miR-335-5p) were less expressed in L-MSCs than in M-MSCs. As previously discussed [5], this observation is in line with other studies [23] as well as with the hypothesis that leiomyoma cells, characterized by an aberrant production of ECM, display a strong interaction with the ECM itself. The expression of miRNAs related to the ECM pathway has been previously analyzed in tissues, fibroblasts, and smooth muscle cells derived from myometrium and leiomyoma [26,27,28], and the results have always highlighted their dysregulation in uterine fibroids. Some previous studies have focused on different miRNAs, such as members of the miR-29 family; however, differentiated cells, instead of mesenchymal cells, were used.

AF-MSCs expressed a discrete number of proteins related to the ECM-receptor interaction; among them, integrins are well known to control many cellular functions through cells/ECM crosstalk, including embryonic development, survival, differentiation, and proliferation [29].

While the expression of the three miRNAs converging in the TGFβ signalling pathway (hsa-miR-122-5p; hsa-miR-576-3p; hsa-miR-595) was weakly altered in M-MSCs compared to AF-MSCs, they were strongly upregulated in L-MSCs.

It has been reported that AF-MSCs express SMAD2/4 (both targeted by hsa-miR-122-5p and hsa-miR-595), as well as NODAL (a target of miRNA hsa-miR-122-5p) and may therefore play a role in regulating self-renewal, similarly as in human embryonic stem cells [30].

The TGFβ pathway is greatly altered in leiomyoma [31,32,33]; even though it is generally described as upregulated in leiomyoma compared to healthy myometrium (and in this line miRNAs converging to this pathway were expected to be downregulated), the TFGβ pathway consists of many different proteins that act with distinct, and sometimes even opposite, effects. Furthermore, upregulated miRNAs in leiomyoma do not target TGFβIII, which is considered one of the main inducers of aberrant ECM production and the reduced level of ECM degradation in uterine fibroids [32]. Indeed, TGFβIII elevated serum level is among the risk factors for the onset of leiomyoma [34]. Previous studies have reported that TGFβIII is one of the very few growth factors detectable at higher concentrations in uterine fibroids [35]. SMAD7 is one of the target proteins of hsa-miR-595 that, by the interaction with TGF-β/activin type I receptors (targeted by hsa-miR-122-5p), can inhibit TGFβ/activin signaling [36]. Taken together, the lack of TGFβIII and the presence of SMAD7 negatively affected by the high expression of hsa-miR-595, may explain the apparent contradiction between the level of expression of the miRNAs and the upregulation of TGFβ signaling observed in leiomyoma.

The cell cycle pathway is strictly related to TGFβ, which acts as a regulator of cell growth. It inhibits the growth of most cell types while stimulating the growth of fibroblasts [37]. The growth-inhibitory response may be a result of the TGFβRI/TGFβRII/SMAD2/3/4 signaling cascade. SMAD2/3/4 complexes (SMAD 4 is targeted by hsa-miR-146a-5p) activate the transcription of CDK inhibitors (such as CDKN1A targeted by hsa-miR-146a-5p, along with other cell division cycle (CDC) proteins, such as CDC23, CDC25A, and CDC25B targeted by hsa-miR-146a-5p), p15, and p21 [38] causing cell cycle arrest [39]. In AF-MSCs, the high expression of these miRNAs and the consequently weak expression of the target proteins correlate with the maintained strong ability to proliferate and to self-renew; the great variability observed in M-MSCs and L-MSCs samples in the expression of miRNAs belonging to cell cycle pathway reflects the highly heterogeneous proliferative capacity reported by Busnelli [40].

Previous studies on the expression profile of miRNA in fibroid and myometrium tissues have identified a list of involved pathways that encompasses the four considered in this study [41].

Interestingly, even though the four pathways are enclosed in the aforementioned list, the converging miRNAs are different. This contradiction may be explained by the well-established fact that multiple miRNAs target the same genes. However, the finding that the same pathways have been found differentially regulated in progenitor as well as in differentiated cells or tissues confirms their involvement in the onset and development of leiomyoma. This hypothesis is also strengthened by the evidence that the selected miRNAs target more proteins belonging to the same pathways and these proteins show a strong interaction network highlighting the fact that miRNAs act with a convergent and synergistic effects. In this complex scenario, our work is not significant as a further confirmation of the involvement of these pathways but rather because it, for the first time, focuses on progenitor cells that, like the differentiated ones, are subjected to a fine regulation through miRNAs expression. It also opens to the evidence that miRNAs act as triggers for the activation of various pathways during differentiation. The role of miRNAs is also evidently linked to the genetic abnormalities, which, together with hormonal and environmental factors, seem to favor the onset of leiomyoma [42]. About 40% of leiomyomas has non-random and tumor-specific chromosomal abnormalities and, consequently, much attention has been paid to the study of genes located in chromosomal regions affected by recurrent changes, such as the subunit 12 of the mediator complex (MED12), high mobility group AT-Hook 2 (HMGA2), and type I procollagen cooh-terminal proteinase enhancer (PCOLCE).

MED12, implicated as an oncogene in about 70% of uterine leiomyoma [43,44], is a target of hsa-miR-10a-5p, whose expression is gradually reduced from AF-MSCS to L-MSCs, possibly explaining the upregulation of MED12 in leiomyoma. HMGA2 plays a key role in the onset of uterine fibroids and resides in the chromosomal rearrangements affecting the 12q14-15 region that targets the HMGA2 gene. It is usually overexpressed in leiomyoma [45] and this is consistent with the lower expression of the relative hsa-miR-10a-3p found in L-MSCs.

PCOLCE maps to the critical interval on chromosome 7, q22band, and a 60% decrease of its expression was found in fibroid tissues compared to myometrial levels in the same patient [46]. PCOLCE is a target of hsa-miR-122-5p whose expression gradually increases from AF-MSCS up to L-MSCs.

## 5. Conclusions

In conclusion, the adherent junction, ECM-receptor interaction, TGFβ, and cell cycle pathways drive the onset and the development of leiomyoma, not only in differentiated cells and tissues as previously reported, but also at the mesenchymal level where their alterations are already detectable.

MiRNAs, acting as switches during the differentiation process, can turn these pathways on or off. However, our results do not trace a precise mechanism underlying the involvement of miRNAs and their mode of action (whether alterations in miRNAs and target pathways occur gradually during the differentiation process or result from divergent cellular engagement).

Further functional analyses are needed to better explain the role played of miRNAs during the differentiation of progenitor cells; once again, miRNAs target the major genes linked to the genetic predisposition to leiomyoma. Despite this growing evidence on the involvement of miRNAs, it is not yet possible to establish their role or whether they act as a causal or consequential effect of a phenomenon.

## Figures and Tables

**Table 1:** Identification of protein targets by mirTarBase software.

KEGG Pathway	miRNA	TARGET	Validation Methods				
			Strong Evidence			Less Strong Evidence	
			Reporter Assay	Western Blot	qPCR	Microarray	NGS
Adherens junction	hsa-miR-10a-5p	ACTG1			•	•	•
		YES1					•
		CTNND1					•
		MAP3K7	•	•	•	•	
	hsa-miR-200a-3p	CTNNB1	•	•	•		
		TCF7L1	•				
ECM- receptor interaction	hsa-miR-335-3p	COL4A1					•
	hsa-miR-335-5p	CD36				•	
		COL6A1				•	
		COL6A5				•	
		COL6A6				•	
		GP9				•	
		HSPG2				•	
		ITGA1				•	
		ITGA10				•	
		ITGA2				•	
		ITGB4				•	
		ITGB5				•	
		ITGB6				•	
		ITGB8				•	
		LAMA5				•	
		LAMB1				•	
		TNC	•		•	•	
		VTN				•	
		THBS3				•	
		SPP1				•	
TGF-β	hsa-miR-122-5p	NODAL				•	
		RBL1					•
		RPS6KB1					•
		SMURF2				•	
Cell cycle	hsa-miR-146a-5p	CDKN1A	•				
		SMAD4	•	•	•	•	

Dots (•) indicate the methods used for the validation of each target.

## Data Availability

The datasets used and/or analysed during the current study are available from the corresponding author on reasonable request.
